# Research on the Analysis of Residual Stress in Heat Treatment of Bellows Using ABAQUS

**DOI:** 10.3390/ma17133263

**Published:** 2024-07-02

**Authors:** Anheng Wang, Chuanwen Ling, Xiang Zhao, Hui Wang, Tao Wang, Guangming Tao, Yanchao Fu, Tao Cheng

**Affiliations:** 1 School of Mechanical and Automotive Engineering, Anhui Polytechnic University, Wuhu 241000, China; cwlsh904@163.com (C.L.); zxahpu@126.com (X.Z.); chenkeyouahpu@163.com (H.W.); ahpuwt@163.com (T.W.); 2 Wuhu Taihe Pipe Insdustry Co., Ltd., Wuhu 241000, China

**Keywords:** bellows, heat treatment, numerical modeling, residual stress, deformation amount

## Abstract

Taking austenitic stainless-steel bellows as the research object, a finite element model for the heat treatment of austenitic stainless-steel bellows was constructed based on ABAQUS CAE 2022. The physical properties of the bellows after the heat treatment were analyzed using experimental and simulated curve processing analysis methods. The changes in residual stress and deformation in relation to the bellows under different cooling times were explored, as well as the distribution of residual stress and deformation at a certain cooling time. The results show that as the cooling time of the heat treatment increases, the residual stress of the bellow decreases significantly, the reduction rate accelerates, and the degree of deformation gradually decreases. When the cooling time of the heat treatment is 900 s, the residual stress of the wave peak in the middle position of the bellow is relatively small, and the residual stress value of the wave valley along the axis direction does not change significantly. The deformation degree of the wave peak and valley axis direction is relatively uniform.

## 1. Introduction

Stainless-steel bellows, as flexible and corrosion-resistant metal processing parts, are widely used in fields such as automobiles, chemicals, and petroleum and natural gas. They play various important roles, including absorbing vibration and displacement, optimizing heat exchange efficiency, and improving design flexibility [1,2]. Residual stress is generated in the bellows during the forming and welding processes [3,4], affecting their corrosion resistance and reducing their fatigue life and mechanical properties [5,6]. It is necessary to reduce the impact of residual stress on bellows through heat treatments [7]. Therefore, studying the residual stress in bellows is becoming increasingly important.

Bellows experience residual stress during welding and forming, and there is currently some research on the analysis of residual stress relating to welding. Lu et al. [8] studied the distribution of residual stress during steel plate welding, established a finite element model, and measured the residual stress relating to welding using an X-ray diffractometer. Guo et al. [9] discussed the distribution of the temperature field and residual stress field during the welding process, as well as residual stress after bulging, through the use of finite element simulation. Mai et al. [10] studied the effects of the welding sequence and interlayer temperature on the temperature and residual stress distribution at different positions of saddle welds by examining the results of numerical simulations.

Heat treatment has a significant impact on residual stress in steel materials, and appropriate heat treatment conditions can effectively reduce or regulate residual stress, thereby improving the material’s properties and crack propagation behavior. Jonan et al. [11] investigated the effect of heat treatment on residual stress in lap-welded steel specimens, revealing that when heat treatment is performed at high temperatures, the initial residual stress value can sometimes decrease by more than 50%. Jae et al. [12] investigated the effect of heat treatment conditions on residual stress in the laser powder bed melting of aged martensitic 18Ni-300 steel. The results showed that when the cantilever specimen was subjected to aging heat treatment, deformation was significantly reduced, indicating a reduction in inherited residual stress. Ji et al. [13] analyzed the formation and evolution mechanism of residual stress during the solid solution heat treatment of 316L/Q235B composite plates, as well as the influence of different factors on residual stress. The results indicate that the bending stress caused by interlayer thermal stress is the main factor for the formation of residual stress in the solution heat treatment process of composite plates. Fan et al. [14] investigated the effect of residual stress caused by three different cooling methods during heat treatment on the crack propagation behavior of GH4169 discs. The results showed that the AJC method suppresses the crack surface opening by introducing residual compressive stress near the center hole surface, thereby improving the crack propagation life of the disc.

There are also explorations and studies focused on the mechanism of residual stress generation and its distribution characteristics. Lin et al. [15] used a cos α X-ray diffraction measurement to verify the effect of forming pressure on residual stress. The results showed that using multi-layer bellows can achieve a lower-forming residual stress. Li et al. [16] used the finite element simulation method to analyze the changes in temperature and stress of steel pipes under different cooling intensities. The results showed that as the cooling intensity increased, the residual stress of the steel pipe gradually increased, and the residual stress was mainly concentrated near the wall of the steel pipe. Wu et al. [17] used the finite element method and indentation test method to study the residual stress distribution on the inner surface, outer surface, and thickness direction of T-joints.

In summary, the existing research has rarely discussed the distribution of residual stress in bellows based on changing the heat treatment conditions. It is challenging for theoretical calculations to meet the requirements of complex working conditions. Finite element numerical simulation can effectively meet practical needs, handle various complex working conditions, and quickly obtain results, reducing the actual testing costs and time [6]. In order to deeply analyze the distribution of residual stress in bellows after heat treatment, as well as the variation law of these stresses under different cooling times, we constructed a numerical model. This model not only considers the unique properties of the material, but also fully combines the geometric characteristics of the bellow. Through the operation and analysis of this model, we successfully obtained a detailed distribution of the residual stress present in the bellows after undergoing heat treatment. This achievement not only reveals the complexity and diversity of stress distributions in bellows during heat treatment but also provides valuable data support for subsequent research. At the same time, the model also simulated the changes in the residual stress of the bellows under different cooling times. This simulation process enables us to directly observe the specific law of the influence of the cooling time on residual stress, providing a powerful theoretical basis for optimizing heat treatment processes and reducing residual stress in relation to bellows.

## 2. Materials and Experiments

### 2.1. Material Performance Parameters

Stainless-steel bellows were welded into circular pipes through thin steel plates, which were then formed using two spinning processes. SUS304 austenitic stainless steel was used, and the chemical composition is shown in Table 1. SUS304 stainless steel is provided by Shanghai Shida Precision Stainless Steel Co., Ltd., Shanghai, China.

The material performance parameters are shown in Table 2.

**Figure 1:**
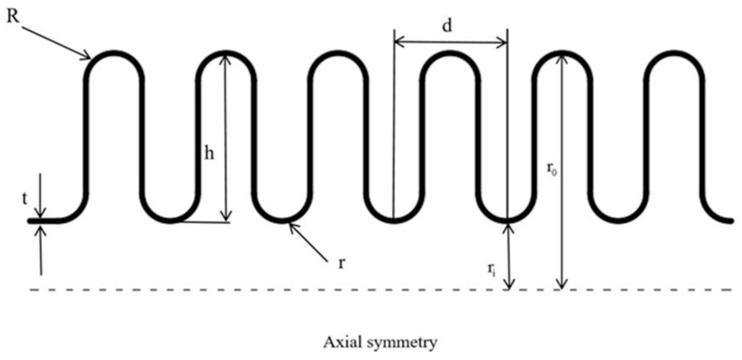
Geometric structure plan of bellows.

Austenitic stainless-steel bellows were the research object. The bellows were formed by spinning, and their structure has axial symmetry. In theory, the waveform of the bellows is highly similar. In order to more clearly represent the shape and structural characteristics of the bellows, a two-dimensional plane diagram was constructed, as shown in Figure 1. The structural parameters of the bellows are shown in Table 3.

### 2.2. Residual Stress Measurement Experiment

#### 2.2.1. Measurement Principle

The testing methods for residual stress can be divided into two categories: destructive testing methods and non-destructive testing methods. The loss testing method measures residual stress through stress release, and commonly used loss testing methods include the blind hole method and the ring core method. Non-destructive testing methods are mainly used to measure residual stress through physical means and do not damage the workpiece. Commonly used non-destructive testing methods include X-ray diffraction, the magnetic method, ultrasonic method, etc. Compared with the above testing methods, both the blind hole method and ring core method can cause damage to the specimen. Furthermore, their operation is complex, and both have low accuracy [18]. The magnetic method is not suitable for austenitic stainless steel; and stresses above two-dimensional stress pose challenges for the ultrasonic method [19]. After more than half a century of development, the X-ray diffraction method for measuring stress has matured and can be used to perform non-destructive testing on various complex workpieces with high accuracy and strong sensitivity [20].

**Figure 2:**
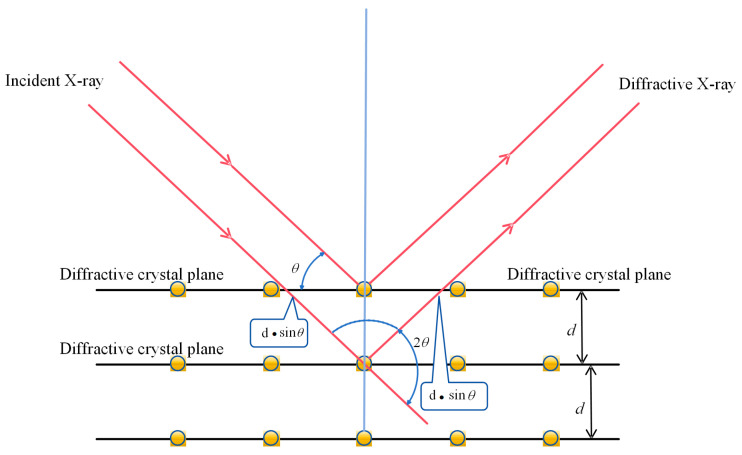
Principle of the X-ray diffraction method.

The basic idea of measuring residual stress using the X-ray diffraction method is to use the diffraction phenomenon of X-rays in a crystal to obtain information about the crystal structure [21]. The principle is shown in Figure 2.

During the measurement process, the known wavelength of an X-ray is first irradiated onto the sample at different incident angles, and then the corresponding diffraction angle is measured [22]. By measuring the change in diffraction angle, the magnitude and distribution of residual stress in the material can be inferred. X-ray stress measurements are based on Bragg’s law [23].

(1) 2d sin⁡θ=nλ

In the formula, *d* represents the interplanar spacing, θ denotes the angle between the incident direction and the reflection plane, *n* indicates the diffraction index, and λ represents the wavelength of the X-ray.

#### 2.2.2. Residual Stress Measurement

**Figure 3:**
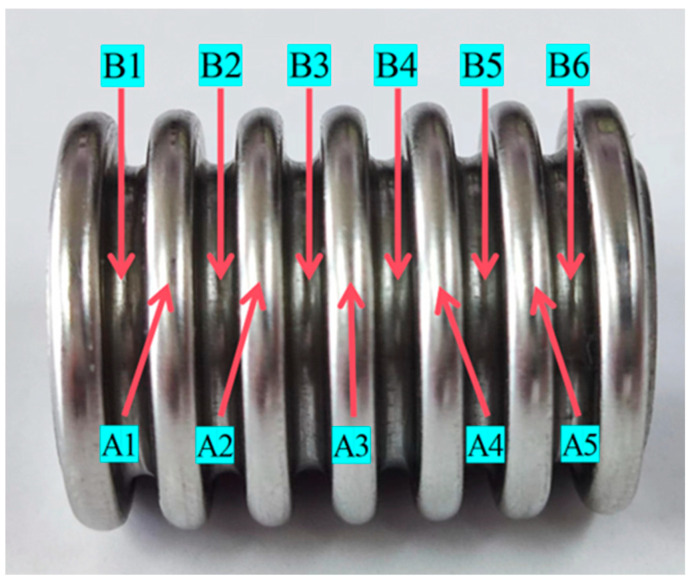
Schematic diagram of residual stress testing points for stainless-steel bellows.

Adopted axial testing of corrugated hoses was carried out, and they were measured using an X-ray residual stress analyzer. The measured sample was a 600 °C aging-treated stainless-steel pipe. Due to the geometric structure of the corrugated pipe and other reasons, the residual stress testing positions were selected from the peak and valley (peak measurement points A1, A2, A3, A4, and A5; valley measurement points B1, B2, B3, B4, B5, and B6) for the axial residual stress measurement. The residual stress measurement positions are shown in Figure 3. The testing equipment included a portable residual stress detection system, with austenitic stainless steel selected as Mn and the X-ray tube as the target material. The tube pressure was 14 kV, and the tube current was 4 mA.

X-rays are electromagnetic waves with short wavelengths that can penetrate a certain thickness of material and undergo diffraction in crystals. When X-rays are irradiated on the surface of a crystal, the grains inside will undergo scattering. During this scattering process, X-rays interact with atoms in the crystal, causing them to change direction. This phenomenon is called diffraction. The angle of diffraction is closely related to the crystal structure and lattice parameters. When the crystal is affected by residual stress, the plane spacing of the crystal plane will change. This change will cause a corresponding shift in the diffraction angle of the incident X-ray. By measuring the change in the diffraction angle and combining it with the Bragg equation, the magnitude and distribution of residual stress in the material can be inferred. During the measurement process, it is necessary to accurately control the incident angle and diffraction angle to conduct effective data analysis and processing.

### 2.3. Micro Organization and Macro Structure

**Figure 4:**
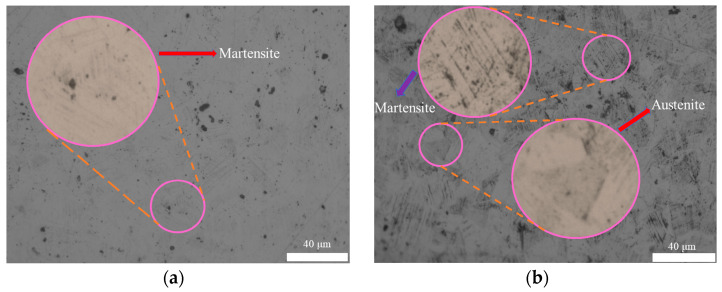
Metallographic structure before and after heat treatment. (**a**) Preheat treatment. (**b**) After heat treatment.

The metallographic structure of stainless-steel bellows before and after heat treatment can visually present the microstructure and grain size of the bellows material [24,25]. As shown in Figure 4, the observation results of the metallographic structure show that after undergoing heat treatment, the internal structure of the bellow significantly transforms into austenite. Austenite, as an excellent microstructure, not only endows bellows with excellent plasticity and toughness, but the formation of austenite also significantly improves the corrosion resistance of bellows. This transformation means that bellows can withstand significant deformation without being easily broken in complex and ever-changing working environments, greatly enhancing their structural stability and reliability.

**Figure 5:**
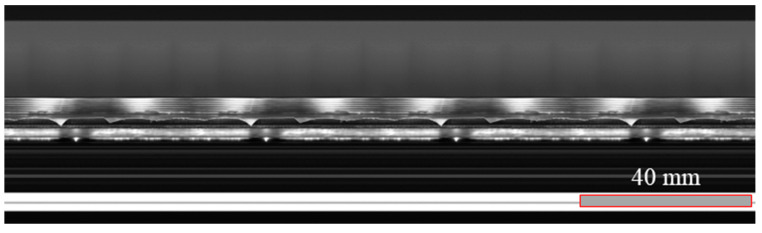
Scanning image of the metal outer surface of the corrugated tube.

Using the Hikvision array camera setup, along with optical cameras and image processing software, as illustrated in Figure 5, it is evident that the corrugated tube exhibits good smoothness and surface quality after undergoing two rounds of spinning and heat treatment.

### 2.4. Mechanism of Residual Stress Generation

During mechanical processing, uneven plastic deformation of the material occurs due to geometric asymmetry or the uneven force distribution of the mechanical shape. After processing, material deformation in different areas can cause relative tension or compression in adjacent areas, resulting in residual stress [26]. Firstly, the bellows were formed by welding steel plates into circular pipes, generating significant and complex residual stresses during the welding process; secondly, the circular tube needs to finally be formed through multiple spinning processes, and significant residual stresses are generated during the forming process.

## 3. Construction of Finite Element Model for Bellows

### 3.1. Geometric Model

**Figure 6:**
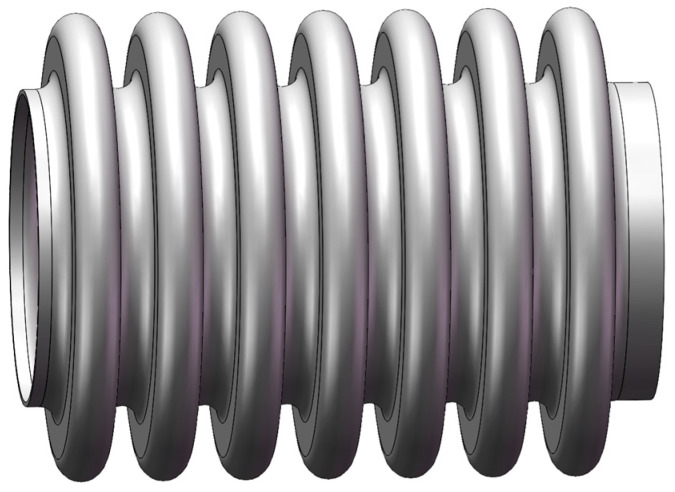
Three-dimensional modeling diagram.

Referring to the structural parameters of the bellow provided in Table 3, we constructed a three-dimensional model as shown in Figure 6.

### 3.2. Grid Division

**Figure 7:**
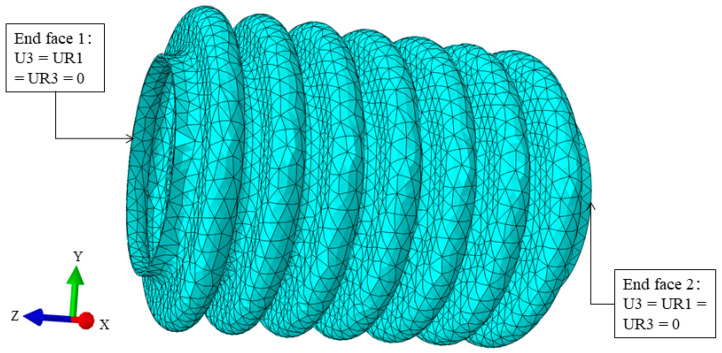
Boundary condition.

After establishing a three-dimensional model of the bellows, mesh partitioning is required. The quality of the grid directly determines the accuracy of finite element simulation and the computational speed of the computer. High-quality grids greatly reduce the iteration time and error probability during the calculation process. Grid partitioning is one of the most important modules in finite element analysis. This article adopted a network partitioning method suitable for irregular models, and the Element Shape adopts a Tet shape, which was selected as the Element Shape. The Tet shape has strong adaptability and is easy to generate. It has significant advantages when dealing with complex geometries and has large deformation capabilities; however, it still has some drawbacks, such as the possibility of causing a high number of grids that will affect the calculation speed and accuracy [27]. To improve the quality of the grid, this article divides the bellows into regions and performs grid division based on segmentation. The approximate global seed size is 0.83, the maximum deviation factor for curvature control is controlled at 0.1, and the minimum size for global percentage is controlled at 0.1. The grid division is shown in Figure 7.

### 3.3. Boundary Conditions

The simulation of the bellow heat treatment included three temperature displacement coupling analysis steps (analysis step 1 applied the heating temperature, analysis step 2 applied the insulation temperature, and analysis step 3 applied the cooling environment temperature). The simulation time for analysis steps 1 and 2 was fixed, at 600 s and 1200 s, respectively. As this article explores bellow heat treatments with different cooling times, the simulation time used in analysis step 3 is a variable: 300 s, 600 s, 900 s, 1200 s, 1500 s, and 1800 s, respectively.

The analysis steps all consider geometric nonlinearity and use fixed time increments in the increments. The actual process of corrugated pipe heat treatment is achieved by controlling the radial deformation of the corrugated pipe by constraining “displacement/rotation”. In these three analysis steps, geometric constraints are on the end faces on both sides of the corrugated pipe. The specific constraint definitions are shown in Table 4 and Figure 7, and geometric constraints are implemented throughout the entire simulation process.

### 3.4. Theoretical Model

This study used 304 stainless steel, and the material deformation behavior was modeled using the Johnson–Cook constitutive model [28], as shown in Equation (2).

The J-C constitutive model comprehensively considers the relationship between the rheological stress and strain, strain rate, and temperature, and can meet the simulation material requirements under various conditions. The J-C constitutive parameter in ABAQUS CAE 2022 is defined by a hardening in the material plasticity, where “Transition Temperature” refers to the yield stress not affected by temperature changes at or below this temperature. When fitting temperature parameters, the turning point of the temperature rheological stress curve can be used to determine and replace the room temperature parameters in the original model, thereby improving the accuracy of the model.

(2) $$\sigma =\left(A+B\varepsilon n\right)\left[1+C\ln(\frac{\varepsilon^{.}}{\varepsilon_{0}^{.}})\right]\left[1-(\frac{T-T_{r}}{T_{m}-T_{r}}{)}^{m}\right]$$

In the formula, σ represents the rheological stress, *A* indicates the yield stress at the reference temperature and strain rate, *B* denotes the strain hardening coefficient of the material, *ε* represents the plastic strain, *n* denotes the strain hardening index of the material, $\varepsilon^{.}$ indicates the strain rate, $\varepsilon_{0}^{.}$ represents the reference strain rate, *T* denotes the test temperature, and $T_{r}$ and $T_{m}$ relate to the room temperature and material melting point.

### 3.5. Simulation of Heat Treatment Conditions

Heat treatment conditions usually involve heating, insulation, and cooling. This article focused on the heat treatment process used in the case of austenitic stainless-steel bellows and explored the residual stress of bellows under different cooling times with the aim of improving the effects of residual stress and surface corrosion [29,30]. During the heating stage, the material was slowly heated to the predetermined temperature, and the heating speed was controlled within a certain range to avoid excessive internal stress or uneven tissue changes caused by excessive heating. The insulation stage was used to maintain the material’s internal structure for a period of time after reaching the predetermined temperature, allowing sufficient time for homogenization and stabilization. The cooling stage is the most critical step in the heat treatment process. An excessive cooling speed may lead to excessive residual stress inside the material, while a slow cooling speed may affect the improved performance of the material.

During the heating stage, the material’s properties are defined, and the thermal conductivity, coefficient of thermal expansion, Young’s modulus, Poisson’s ratio, and other parameters of the material are set, which are used to establish the thermal elastic constitutive relationship of the material. A heat conduction equation is established. Based on the heat conduction properties of the materials and the geometric shape of the structure, a heat conduction equation was established to describe the temperature field distribution of the structure. To define the boundary conditions and loading conditions, the heating boundary conditions were set; the outer surface of the bellow was subjected to a high temperature, and the inner surface was subjected to room temperature. Concerning mesh partitioning, the structure was discretized into a finite element mesh.

During the cooling stage, the cooling conditions are defined as follows: the boundary conditions for cooling are determined, the boundary temperature of the structure is changed, and the cooling process is simulated. For the numerical solution, the same finite element model and solution method are used to solve the temperature field during the cooling process.

## 4. Analysis of Numerical Simulation Results

### 4.1. Distribution of Residual Stress under Different Cooling Times

During the bellow heat treatment process, different cooling times can lead to different internal stress distributions. The direct impact of the cooling speed on the temperature difference between the surface and the center of the bellows can cause uneven volume expansion and contraction, resulting in internal stress. Finite element numerical simulation was used to analyze the residual stress distribution on the surface of the bellows with different cooling times without changing the heating and insulation stages. This simulation analyzed the residual stress of the bellows after heat treatment under cooling times of 300 s, 600 s, 900 s, 1200 s, 1500 s, and 1800 s. The results of the analysis are shown in Table 5.

#### 4.1.1. Evolution Law of Residual Stress Distribution

**Figure 8:**
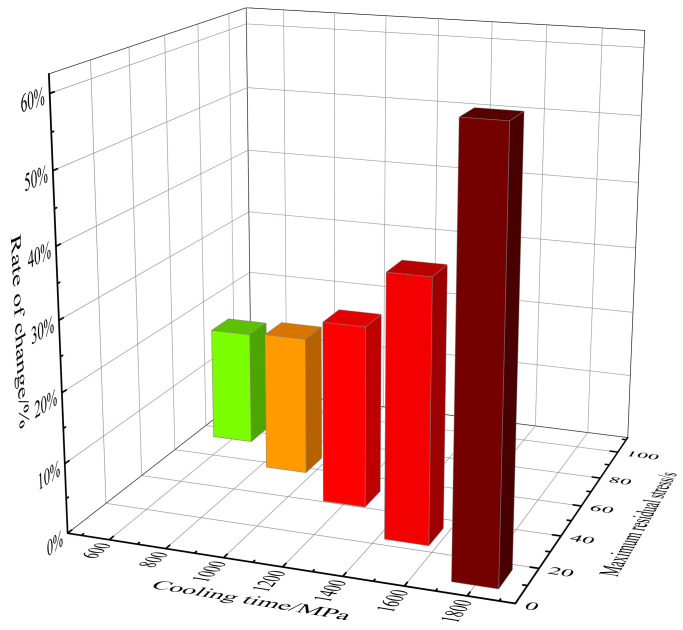
Degree of residual stress reduction under different cooling times.

**Figure 9:**
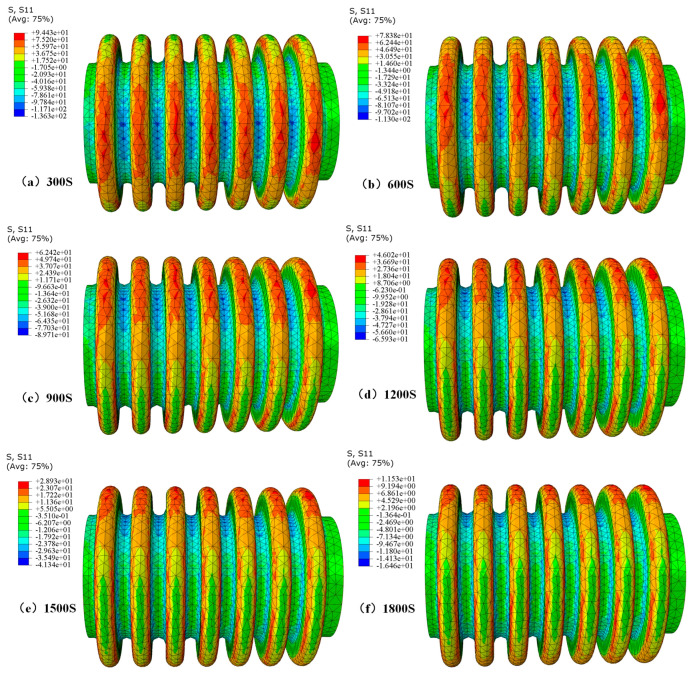
Stress distribution cloud map under different cooling times.

In the case of bellows, the distribution of residual stress is mainly caused by uneven displacement and expansion. Figure 8 shows the bar graph of the residual stress reduction rate under different cooling times. It can be observed that the residual stress reduction rate increases with the increasing cooling time. Figure 9 shows the residual stress distribution cloud map under different cooling times, indicating significant residual stress at the ridge. After data calculation, it can be concluded that in the five cooling time zones of 300 s~600 s, 600 s~900 s, 900 s~1200 s, 1200 s~1500 s, and 1500 s~1800 s, the average residual stress changes per 100 s are 5.35 MPa, 5.32 MPa, 5.47 MPa, 5.70 MPa, and 5.80 MPa. Therefore, it can be concluded that the longer the cooling time, the greater the reduction in residual stress. It should be noted that, for example, in Figure 9 (+9.443e+01), it is expressed as 9.443 × 10 using the Scientific notation.

#### 4.1.2. The Trend of Residual Stress Variation on the Wave Crest

**Figure 10:**
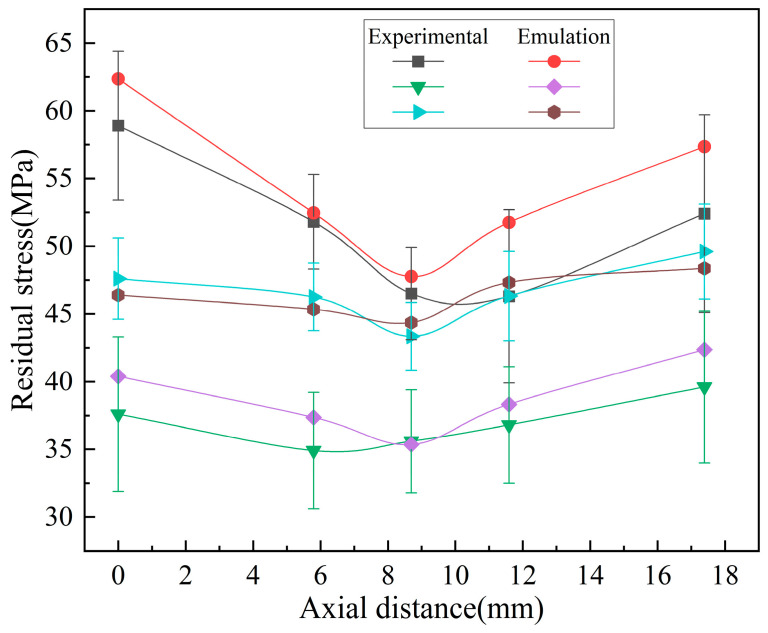
Distribution curve of axial residual stress at the ridge of the wave.

With a cooling time of 900 s, multiple sets of residual stresses along the axial direction of the bellow’s peak are taken. The experimental data and simulation data were compared, as shown in Figure 10. It can be seen that the distribution trend of the residual stresses on the outer surface of the axial wave peak of the bellow after heat treatment is relatively small at the middle position.

#### 4.1.3. The Trend of Residual Stress Variation on the Trough

**Figure 11:**
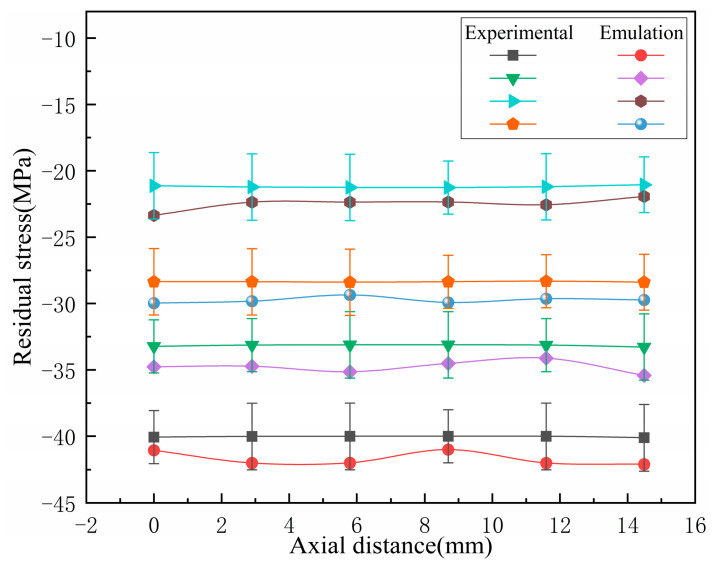
Distribution curve of residual stress in the axial direction of the trough.

With a cooling time of 900 s, multiple sets of residual stresses along the axial direction of the bellow’s trough are taken. The experimental data and simulation data were compared, as shown in Figure 11. It can be seen that the distribution trend of the residual stresses on the outer surface of the axial trough of the bellow after heat treatment is relatively gentle.

### 4.2. The Influence of Different Cooling Times on the Deformation of Bellows

Cooling speed is one of the key factors affecting the rotational displacement of bellows. When cooling, the outer surface of the bellows may first come into contact with low temperatures, which may cause rapid surface shrinkage, while the internal contact may result in a slower cooling speed and contraction. This uneven shrinkage may lead to deformation. When the cooling speed is fast, the temperature difference between the inside and outside of the bellows is large, and the thermal stress is high, which may cause significant deformation during the cooling process. This study used cooling times of 300 s, 600 s, 900 s, 1200 s, 1500 s, and 1800 s to analyze the deformation trends after the heat treatment. The results of the analysis are shown in Table 6.

#### 4.2.1. Deformation Evolution Law of Bellows

**Figure 12:**
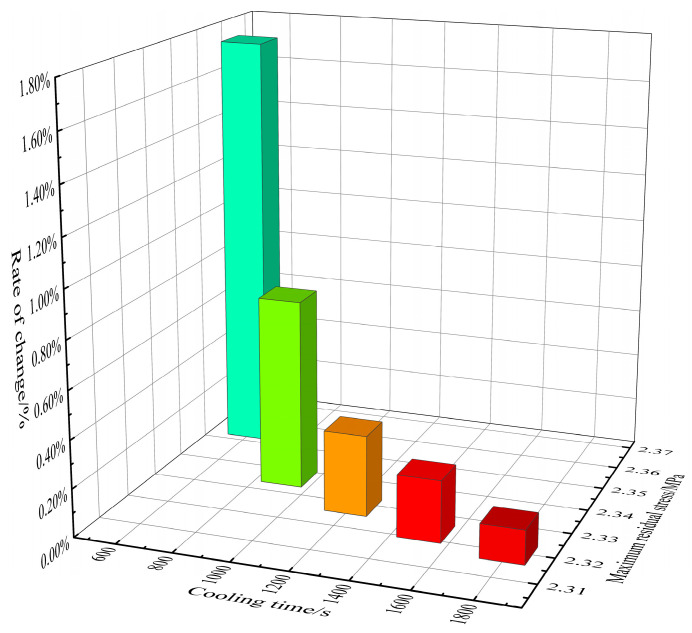
Deformation degree under different cooling times.

**Figure 13:**
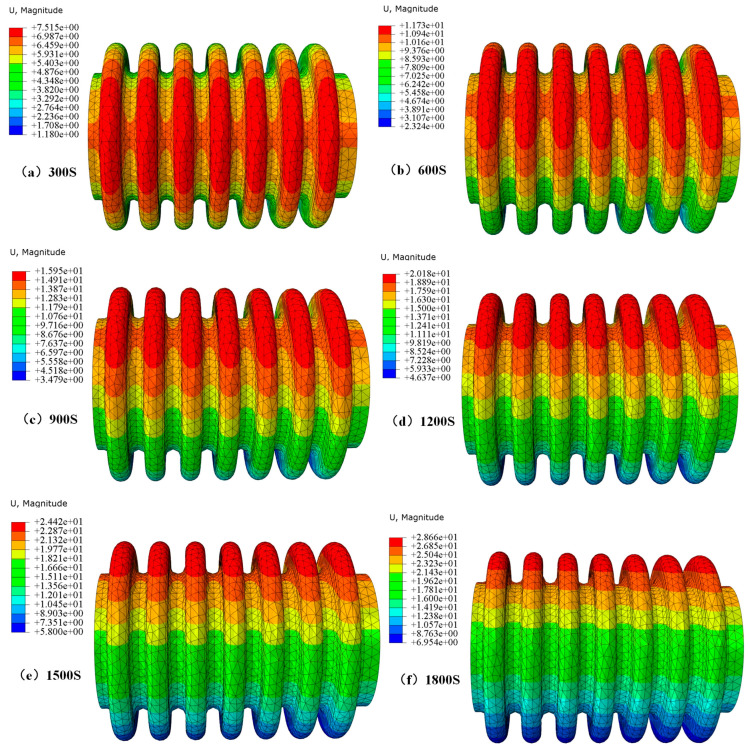
Cloud map of bellows deformation under different cooling times.

The cooling rate of the heat treatment will affect the degree of deformation. Figure 12 shows the deformation curve under different cooling times, and Figure 13 shows the deformation cloud map in relation to different cooling times.

Through finite element simulation of heat treatment cooling conditions, bellow deformation under different cooling rates was obtained. Table 6 shows that even though the deformation change at different cooling rates is negligible, there is still a certain pattern, that is, as the cooling rate slows down, the displacement deformation also slows down. As shown in Figure 12, as the cooling time increases, the degree of residual deformation gradually decreases. As the cooling time increases, the total deformation increases.

As shown in Figure 13, the deformation ratio factors for 300 S, 600 S, 900 S, 1200 S, 1500 S, and 1800 S are 0.3109, 0.2088, 0.1465, 0.1154, 0.09512, and 0.08093, respectively. It should be noted that, for example, in Figure 13 (+7.515e+00), it is expressed as 7.515 using the Scientific notation.

#### 4.2.2. Axial Deformation Trend of Bellows

**Figure 14:**
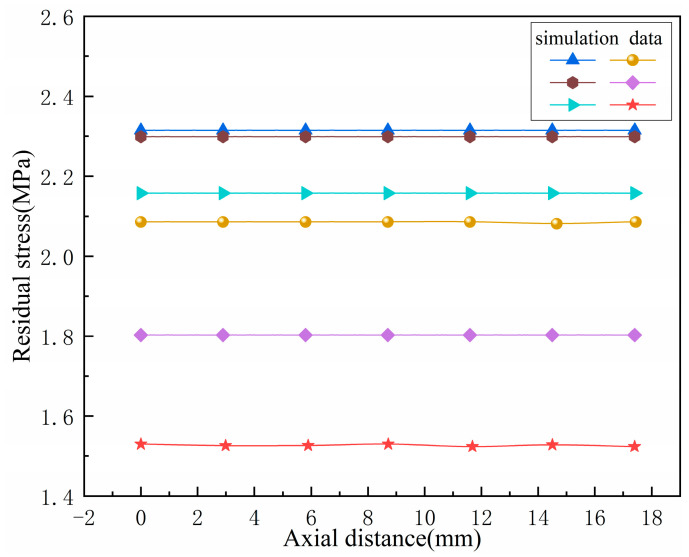
Axial wave ridge deformation curve.

**Figure 15:**
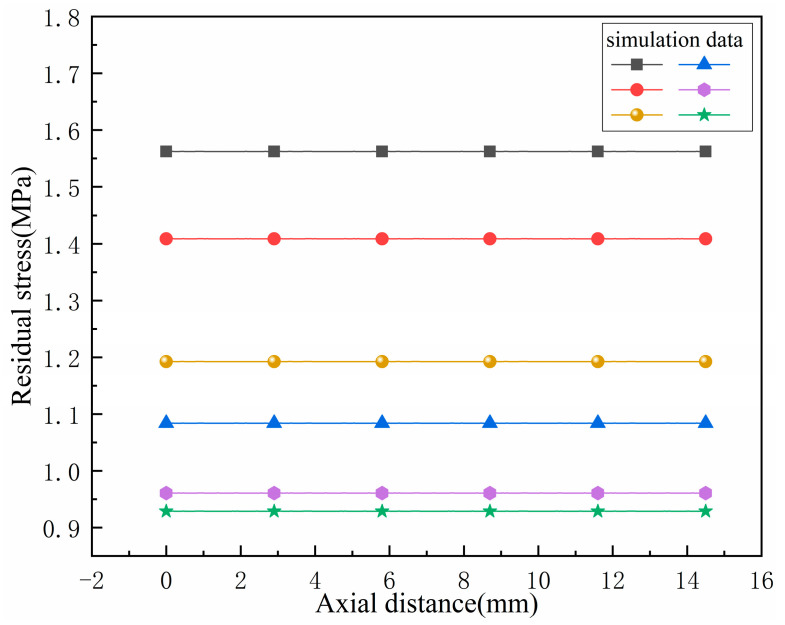
Axial valley deformation curve.

With a cooling time of 900 s, multiple sets of axial wave peak and valley deformations relating to the bellow are taken, as shown in Figure 14 and Figure 15. It can be seen that the axial wave peak and valley deformations of the bellow are relatively uniform after undergoing the heat treatment.

This section explores the deformation of bellows under different cooling times during the heat treatment process through the use of simulation technology. Not only did we observe the deformation phenomenon in detail, but we also further analyzed the specific trend of axial deformation in the case of the bellows and achieved a series of results. However, given the limitations of simulation research, we recognize the importance of experimental validation. Therefore, in subsequent research, we plan to further verify these simulation results via experimental means and explore the specific impact of deformation on the physical properties of bellows, in order to obtain a more comprehensive and accurate understanding.

## 5. Conclusions

Stainless-steel bellows were the research object of this study, and combined with finite element simulation and experimental analysis, the distribution of residual stress in the bellows after heat treatment was studied, as well as the trend of residual stress and deformation when using different cooling times. The conclusions are as follows.

(1) A residual stress analysis model for stainless-steel bellows during heat treatment was constructed using ABAQUS CAE 2022 software. The distribution trends of residual stress and deformation in the bellows during the heat treatment cooling stage and after heat treatment under different cooling time conditions were explored.
(2) Based on numerical simulation, it was found that the residual stress on the surface of the bellows under different cooling times decreases with the increased cooling time. The cooling time starts from 300 s, and the percentage of residual stress reduction for each additional 300 s is 17.00%, 20.36%, 26.27%, 37.14%, and 60.15%, respectively, showing an upward trend.
(3) The analysis of the experimental and simulation results shows that the peak residual stress of the bellow in the middle position is significantly reduced after heat treatment, and the distribution trend of the residual stress in the valley is more uniform. This helps to reduce the risk of stress concentration and stress corrosion cracking, thereby improving the fatigue life of bellows.
(4) The different cooling times of heat treatment have a certain impact on the deformation of bellows, but the impact is relatively small. As the cooling time increases, the formation decreases. Starting from 300 s, for every additional 300 s on the original basis, the deformation decreases by 1.748%, 0.794%, 0.338%, 0.258%, and 0.142%, respectively. The deformation at the peaks and valleys of the bellow is relatively evenly distributed along the axis direction, avoiding the structural instability caused by local stress concentration, making the bellow more resistant to external pressure and impact during use, thus extending its service life.

## Figures and Tables

**Table 1:** Chemical composition of 304 austenitic stainless steel (%, mass fraction).

Element	C	Si	Mn	P	S	Cr	Ni	Other
Content	0.043	0.41	1.07	0.030	0.004	18.01	8.03	0.002

**Table 2:** The 304 performance parameters.

Performance Parameter	Elastic Modulus/GPa	Poisson’s Ratio	Yield Strength/MPa	Tensile Strength/MPa	Elongation Rate/%	HV
Numerical value	197	0.3	267	738	68.0	160

**Table 3:** Structural parameters of bellows.

External Diameter r_0_/mm	Internal Diameter r_i_/mm	Ridge Radius R/mm	Valley Radius r/mm	Wave Distance d/mm	Wall Thickness t/mm	Wave Number	Number of Plies
∅14.9	∅10.8	0.8	0.65	2.9	0.16	7	1

**Table 4:** Definition of model constraints.

Constraint	End Face 1	End Face 2
Displacement (mm)	Z = 0	Z = 0
Rotation (rad)	XY = 0	XY = 0

**Table 5:** Maximum residual stress under different cooling times.

Cooling Time/s	300	600	900	1200	1500	1800
Maximum residual stress/MPa	94.43	78.38	62.42	46.02	28.93	11.53

**Table 6:** Maximum deformation under different cooling times.

Cooling Time/s	300	600	900	1200	1500	1800
Maximum deformation/mm	2.3973	2.3554	2.3367	2.3288	2.3228	2.3195

## Data Availability

Data are contained within the article.
